# Feasibility and effect of interactive telerehabilitation on balance in individuals with chronic stroke: a pilot study

**DOI:** 10.1186/s12984-021-00866-8

**Published:** 2021-04-26

**Authors:** Shih-Ching Chen, Chueh-Ho Lin, Sheng-Wen Su, Yu-Tai Chang, Chien-Hung Lai

**Affiliations:** 1 Department of Physical Medicine and Rehabilitation, School of Medicine, College of Medicine, Taipei Medical University, No. 252, Wu-Hsing St., Taipei City, 110 Taiwan; 2 Department of Physical Medicine and Rehabilitation, Taipei Medical University Hospital, Taipei, Taiwan; 3 Taipei Neuroscience Institute, Taipei Medical University, Taipei, Taiwan; 4 Masters Program in Long-Term Care & School of Gerontology Health Management, College of Nursing, Taipei Medical University, Taipei, Taiwan

**Keywords:** Telerehabilitation, Stroke, Balance, Berg Balance Scale, Randomized controlled trial

## Abstract

**Background:**

Stroke survivors need continuing exercise intervention to maintain functional status. This study assessed the feasibility and efficacy of an interactive telerehabilitation exergaming system to improve balance in individuals with chronic stroke, compared to conventional one-on-one rehabilitation.

**Methods:**

In this prospective case–control pilot study, 30 Taiwanese individuals with chronic stroke were enrolled and randomly allocated to an experimental group and a control group. All participants received intervention 3 times per week for 4 weeks in the study hospital. The experiment group underwent telerehabilitation using a Kinect camera-based interactive telerehabilitation system in an independent room to simulate home environment. In contrast, the control group received conventional one-on-one physiotherapy in a dedicated rehabilitation area. The effectiveness of interactive telerehabilitation in improving balance in stroke survivors was evaluated by comparing outcomes between the two groups. The primary outcome was Berg Balance Scale (BBS) scores. Secondary outcomes were performance of the Timed Up and Go (TUG) test, Modified Falls Efficacy Scale, Motricity Index, and Functional Ambulation Category.

**Results:**

Comparison of outcomes between experimental and control groups revealed no significant differences between groups at baseline and post-intervention for all outcome measures. However, BBS scores improved significantly in both groups (control group: p = 0.01, effect size = 0.49; experimental group: p = 0.01, effect size = 0.70). Completion times of TUG tests also improved significantly in the experimental group (p = 0.005, effect size = 0.70).

**Conclusion:**

The Kinect camera-based interactive telerehabilitation system demonstrates superior or equal efficacy compared to conventional one-on-one physiotherapy for improving balance in individuals with chronic stroke.

*Trial registration* ClinicalTrials.gov. NCT03698357. Registered October 4, 2018, retrospectively registered.

**Supplementary Information:**

The online version contains supplementary material available at 10.1186/s12984-021-00866-8.

## Background

Stroke is a leading cause of disability that imposes a substantial burden on the daily life of those who survive [1, 2]. Disability in individuals with chronic stroke may include poor balance, impaired mobility, limited physical activity, and limited ability to perform activities of daily living, all of which are more common in the acute stage but also may persist for years [3–5]. Muscle weakness in the lower limbs, abnormal muscle tone, sensory deficiency, and abnormalities in vision and spatial awareness may result in deficits in mobility and balance, thereby increasing the risk of falls [6, 7], and leading to serious complications (e.g., hip fractures) [8]. Therefore, it becomes critical to increase physical activity and improve balance, muscle strength and mobility in this patient population.

For stroke survivors, total recovery from disability is not guaranteed after restorative rehabilitation in the acute and subacute stages of stroke [9]. To maximize functional recovery and maintenance of function, patients with chronic stroke must undergo continual rehabilitation or exercise intervention. However, stroke survivors’ willingness to consistently adhere to outpatient therapy is affected by several factors, such as therapist availability, program costs, family support, and access to transportation [10, 11]. Hence, home-based telerehabilitation may help stroke survivors maintain longitudinal continuity of exercise rehabilitation at home, and help reduce therapist workload [12].

Conventional balance and gait physiotherapy techniques include Bobath Concept therapy, motor relearning, and proprioceptive neuromuscular facilitation [13–15], and the efficacy of physiotherapy in improving function in individuals with stroke has been demonstrated. [16–18]. However, these treatments are preferably undertaken in specialized rehabilitation units, which are not available in all hospitals [19]. In addition, rates of adherence to such interventions are typically low because conventional exercises tend to be repetitive and unattractive [20].

In addition, telerehabilitation offers stroke survivors a flexible exercise schedule, allowing them to perform repetitive task-specific training in an independent area of the hospital or at home [21]. Virtual reality (VR) and exergaming can provide real-time augmented feedback while an individual performs specific motor tasks [14, 22]. This feedback on performance offers individuals useful information to help them recreate a sense of body position in space [23]. Moreover, real-time visual and auditory biofeedback enhanced motor learning while individuals perform specific motor tasks as directed [24–26].

Interactive self-rehabilitation programs and exergaming telerehabilitation may allow individuals with chronic stroke to continue exercise training at home with therapists’ remote guidance [27, 28]. Several studies have revealed that self-rehabilitation or home-based interactive telerehabilitation improves upper limb motor function status in individuals with chronic stroke [27, 29, 30]. Some of these home-based or self-rehabilitation exercise programs have been designed to limit therapists’ involvement [28, 29]. Held et al. [28] revealed that those with chronic stroke can undergo on average 71% of scheduled activities without therapists’ involvement. Natta et al. [29] indicated that all 12 individuals in their study of chronic stroke were able to perform the entire self-rehabilitation program effectively. We hypothesized that compared to one-on-one conventional rehabilitation delivered by therapists, interactive telerehabilitation with limited therapist’s verbal engagement has similar effectiveness in improving balance in individuals with chronic stroke. The present study aimed to examine the feasibility and effectiveness of the interactive telerehabilitation exergaming program on balance in individuals with chronic stroke compared to the effects of conventional one-on-one physiotherapy.

## Methods

### Study design and participants

In this prospective case-controlled pilot study, consecutive Taiwanese individuals with chronic stroke were screened in the outpatient clinics of Taipei Medical University Hospital, and were then randomly allocated to an experimental group or a control group. Inclusion criteria were: patients with first-time stroke, motor deficits (hemiplegia or hemiparesis), Brunnstrom stage between II and V, stroke onset time over 6 months, and having no obvious psychological or emotional problems. Brunnstrom recovery stages are defined as: I) Evidence of flaccidity; II) Increased resistance due to spasticity and limb synergies performed voluntarily; III) Spasticity begins to develop; IV) Spasticity is less evident than stage III; V) Minimal resistance from spasticity, and individual as well as complex movement combinations are possible independent of synergy; VI) Spasticity is difficult to demonstrate unless movements are performed with rapidity, and synergies do not interfere with performance [31]. Exclusion criteria were: having a Modified Ashworth Scale of > 3 points, language difficulties or aphasia, severe hearing or visual impairment, or history of severe cardiopulmonary disease.

### Ethical considerations

The study protocol was reviewed and approved by the Joint Institutional Review Board of Taipei Medical University (TMU-JIRB N201708024). The potential risks and benefits of participation in this study were explained to each participant in advance, and all participants provided signed informed consent before participation. The study is registered at ClinicalTrials.gov (NCT 03,698,357).

### Interactive telerehabilitation exergaming system

**Fig. 1:**
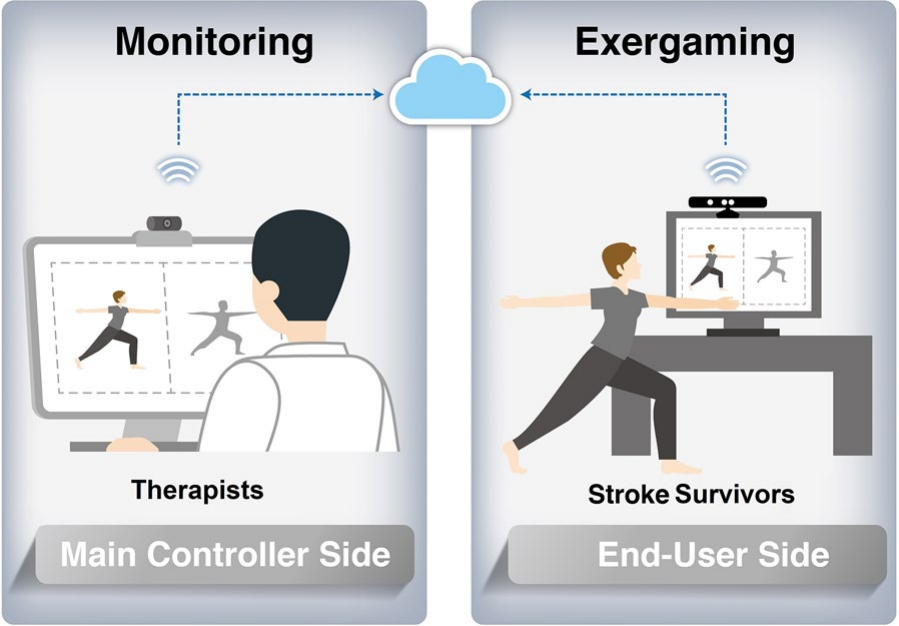
Architecture of the bidirectional telerehabilitation system. This system links a main controller side for therapists and an end-user side for stroke survivors with chronic stroke by wireless sensor network technologies. Both sides have a peripheral component interconnect (PCI) network card. A cloud database center allows therapist to monitor the gaming exercises by stroke survivors instantly or review performance data later

Although all components are commercially available, the whole telerehabilitation system was assembled for the first time by our group. The present system consists of a main monitoring controller for therapists and several connected end-user applications for stroke survivors (Fig. 1). The main controller on the therapist side and the end-user side for participants are linked by wireless sensor network technologies. Both sides have a peripheral component interconnect (PCI) network card. A cloud database center allows therapists to remotely monitor the gaming exercises by stroke survivors in real time or review performance data later.

#### Main monitoring controller

The main monitoring controller allows therapists to monitor exercise accuracy and the safety of individuals with chronic stroke. The end-user applications give users access to visual feedback and game-based programs during training. The training status and associated data are reported to therapists.

#### End-user exergaming applications

The end-user exergaming applications employ a screen and a Kinect sensor (Microsoft Corporation, Redmond, WA, USA). The Kinect sensor incorporates infrared light and a video camera, which create a three-dimensional (3D) picture of the area in front of it. This device enables full-body 3D motion capture. Participants interact with video avatars according to representations on the television screen. The game-based telerehabilitation programs provide goal-directed exercises—that correspond to the participant’s ability—in a virtual environment. The computer calculates the accuracy of the user’s performance or avatar-mimicking trajectory and provides the end user with a specific score. The scores and completion time of the exercises are displayed on the monitor in real time during training and summarized at the end of the exercise. Participants in the experimental group undertook telerehabilitation with remote therapist supervision in an independent room of the hospital to simulate the home environment. Participants were able to independently operate the gaming exercises, because no expert computer skills were required. Family members or caregivers stood beside participants to help prevent falls.

### Telerehabilitation programs

**Fig. 2:**
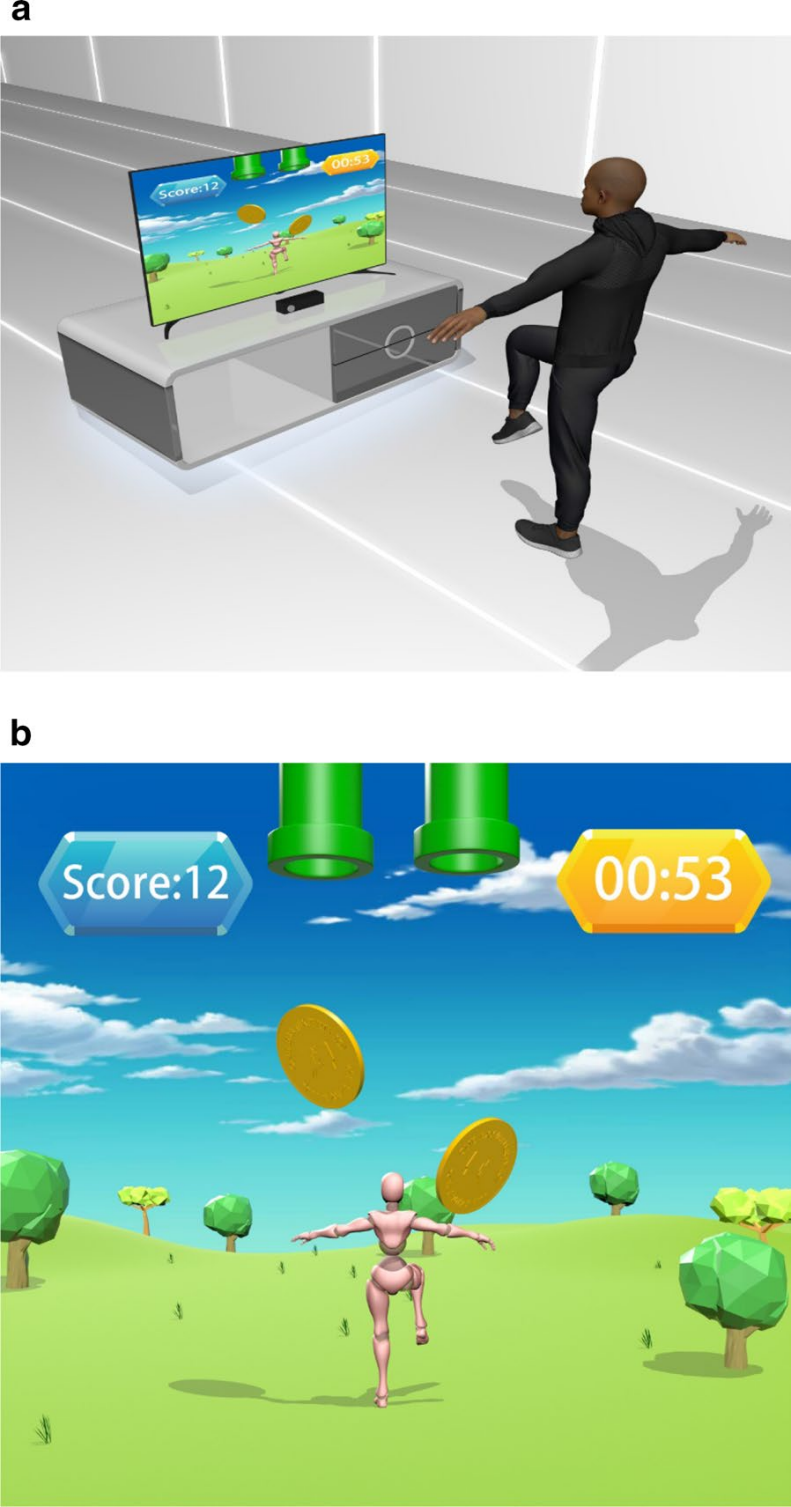
Design of the target-oriented stepping task. Participants underwent the interactive target-oriented stepping task according to the falling objects on the television screen (**a**). Participants raised their legs according to the falling objects on the monitor (**b**)

**Fig. 3:**
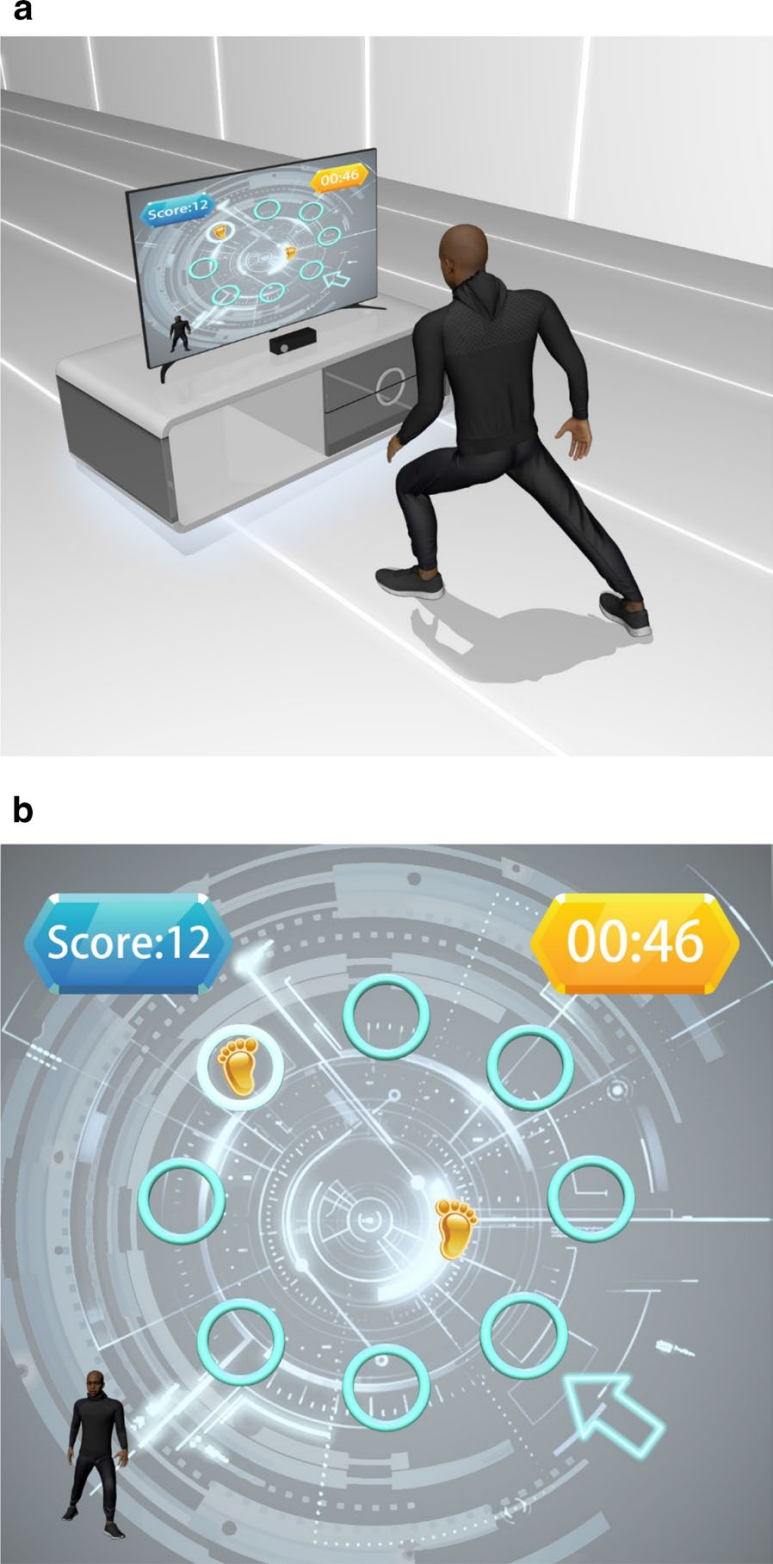
Diagram of the multidirectional reaching task. Participants performed the multidirectional reaching task in front of the monitor (**a**). They were instructed to reach toward a given target in eight directions according to a random cue (**b**)

**Fig. 4:**
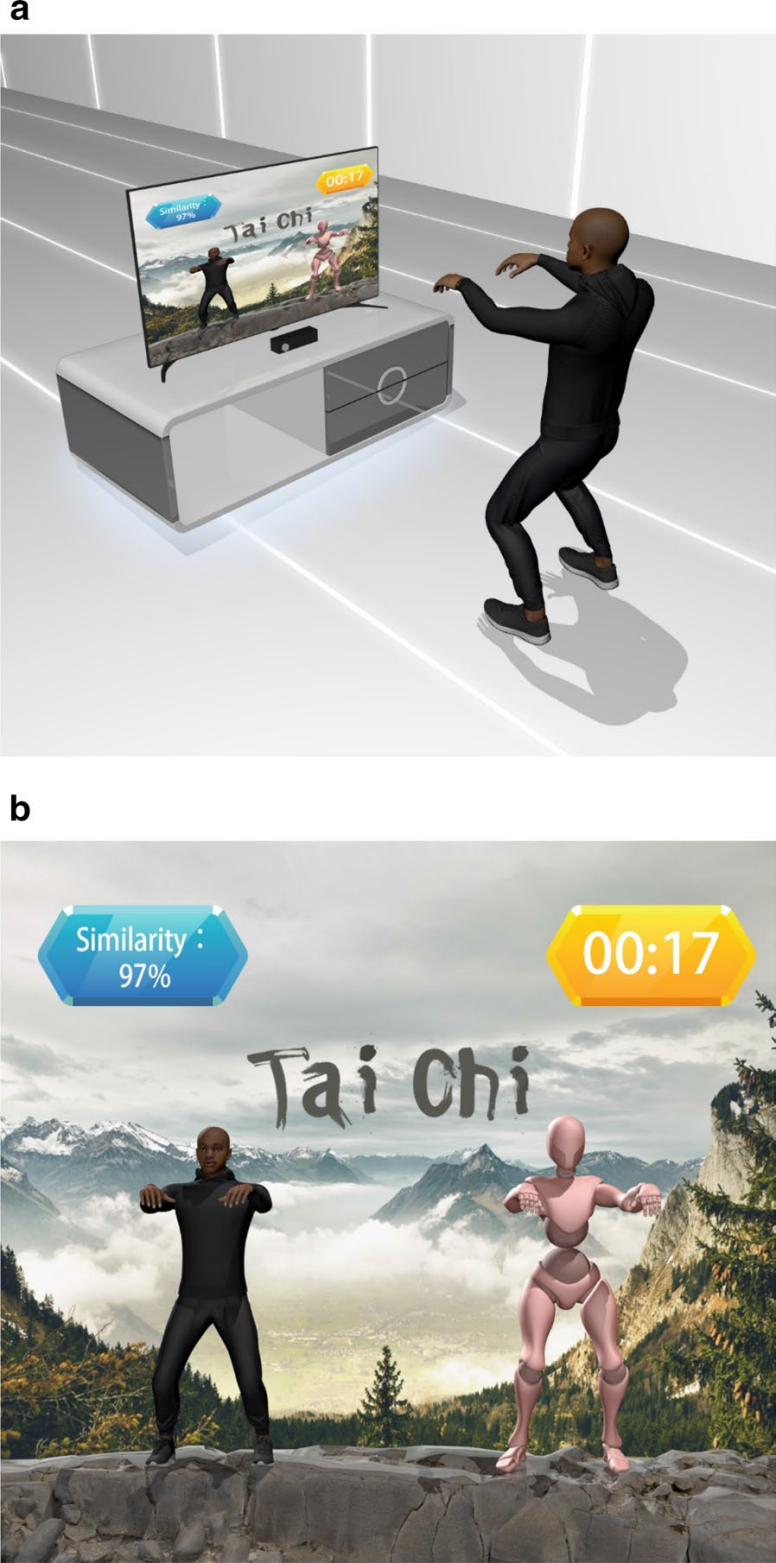
Design of the Tai Chi exercises. Participants underwent the interactive Tai Chi exercises by imitating motions of avatar on the screen (**a**). Avatar representations of participants’ movements were displayed on the monitor (**b**)

For the telerehabilitation program, we chose three commercially available video games (LongGood Co., Ltd, Taipei, Taiwan), focusing on participants’ balance, weight bearing, strength, weight shifting, and walking. The first program is a target-oriented stepping task that trains balance, muscle strength and endurance. Participants are required to lift the paretic leg to a certain height, matching the falling objects on the screen (Fig. 2). The second program is a multidirectional reaching task that focuses on dynamic standing balance and coordination (Fig. 3). Participants were instructed to reach toward a given target in eight directions according to the randomized cue on screen. The third program comprises Tai Chi exercises. These exercises focus on dynamic standing balance, muscle strengthening, and flexibility. Participants follow the action of an avatar on the screen (Fig. 4). A systematic meta-analysis by Li et al. [32] noted that Tai Chi exercises might benefit the gait ability of those with chronic stroke.

### Rehabilitation program performance by group

**Fig. 5:**
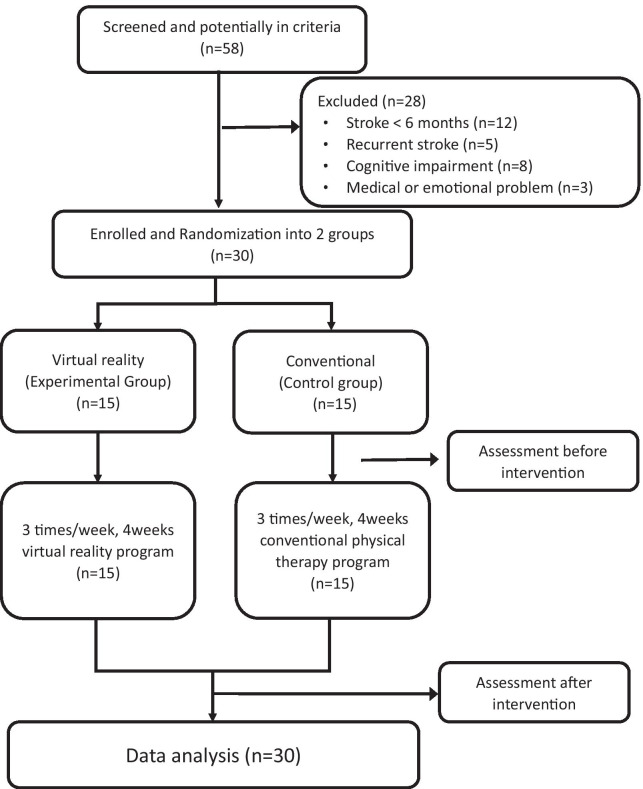
Experimental flow diagram. Thirty participants with chronic stroke were randomly assigned to experimental and control groups. Participant performance was assessed before and after the 4-week intervention

The experimental group participated in 12 interactive telerehabilitation exercise sessions, 40 min per session, including a warm up (5 min), a target-oriented stepping task (10 min), a multidirectional reaching task (10 min), Tai Chi exercises (10 min), and a cool down (5 min), three times a week for 4 weeks (Fig. 5). For the purpose of the pilot study, the program was conducted in an independent room in the hospital rehabilitation department simulating a home-based environment.

Those in the control group underwent 12 one-on-one conventional training sessions that consisted of a warm up (5 min), conventional one-on-one physiotherapy (30 min), and a cool down (5 min) three times a week for 4 weeks in the dedicated rehabilitation facility of the hospital (Fig. 5). The conventional physiotherapy sessions included sitting to standing movements, balance exercises, standing, overground walking, and facilitation or strengthening of the paretic limb. Therapists adjusted the conventional physiotherapy according to the functional status of each participant. Outcome variables were later analyzed by the therapist who was blinded to group assignments.

### Primary outcome measures

#### Berg Balance Scale

The Berg Balance Scale (BBS) includes a series of 14 functional balance tasks to examine balance status in different postures, including maintaining a quiet stance, sitting-to-standing, shifting weight and reaching, turning in place, standing on one leg, and maintaining a tandem stance [33]. Each task is scored on a 5-point ordinal scale (from 0 to 4). A score of 0 denotes inability to perform the task, and a score of 4 denotes the ability to complete the task according to a preset criterion. The maximum score is 56. The interrater and intrarater reliability of the BBS were excellent (ICC 0.98 and ICC 0.97, respectively) in older adults and patients with stroke [34].

### Secondary outcome measures

#### Timed up and go test

The Timed Up and Go (TUG) test is commonly used to examine functional mobility, balance, and fall risk [33, 35]. A cone was placed 3 m from the front of a chair and participants were asked to stand, walk 3 m to the cone, turn around the cone, walk back, and sit down. TUG scores correlated significantly with BBS scores, and demonstrated excellent intrarater, interrater, and test–retest reliability in individuals with chronic stroke [36].

#### Modified falls efficacy scale

Tinetti et al. [37] developed the original 10-activity Falls Efficacy Scale (FES) to assess the degree of perceived efficacy (i.e., self-confidence) at avoiding a fall during each of 10 relatively nonhazardous activities of daily living, focusing on fear of falling while performing exclusively indoor activities. The modified FES (MFES) designed by Hill et al. [38] expands the FES by adding 4 items that question the individual's confidence in performing outdoor activities. The MFES is used to assess the confidence of older adults of not falling while performing a broad scope of daily activities. Items are rated from 0 (not confident at all) to 10 (completely confident), and the maximum score is 140. The MFES has high test–retest reliability (interclass correlation coefficients: 0.93) and internal consistency (Cronbach’s alpha: 0.95) [38], and has been used effectively to assess balance status in people with chronic stroke [39].

#### Motricity index

The Motricity Index (MI) is a reliable method for evaluating paralysis in patients with stroke. The MI was used for classifying the strength in each of three lower extremity muscle actions (hip flexion, knee extension and ankle dorsiflexion) in 6 stages as follows: 0 = No movement; 9 = Palpable contraction, but no movement; 14 = Movement, but not full range or against gravity; 19 = Movement, full range against gravity, not against resistance; 25 = Movement against resistance, weaker than contralateral side; 33 = Normal strength [40]. A total score for paretic lower extremity is obtained by adding one to the sum of the scores of all three individual actions (maximum score: 100). Lower extremity strength scores of the MI were validated previously [40].

#### Functional ambulation category

The Functional Ambulation Category (FAC) is a commonly used clinical gait assessment scale with 6 levels of walking ability based on the amount of.

physical support required, as follows: 0 = nonfunctional ambulatory; 1 = ambulator, dependent on physical assistance [Level II, Manual contacts are continuous and necessary to support body weight and maintain balance]; 2 = ambulator, dependent on physical assistance [Level I, Manual contacts consists of continuous or intermittent light touch to assist balance or coordination]; 3 = ambulator, dependent on supervision; 4 = ambulator, independent, level surface only; 5 = ambulator, independent [41, 42]. The FAC is a quick, easy-to-use previously validated visual measurement of walking and easy for therapists to interpret [42].

### Statistical analysis

Continuous variables are presented as the median and interquartile range and between-group differences were evaluated using the Mann–Whitney U test. Categorical variables are presented as numbers and percentages, and their distribution between groups was examined by chi-square test or Fisher’s exact test. The Wilcoxon signed-rank test was used to evaluate differences between pre-intervention and post-intervention measurements in both groups. Two-way repeated measures analysis of variance (ANOVA) was used to evaluate the differences between groups. The significance level was set as two-sided p < 0.05. For any statistically significant result, its effect size was then calculated to measure the magnitude of the interventional effect. Effect size was calculated by dividing the test statistic by the square root of the number of observations, as previously described [43].

## Results

Among a total of 58 Taiwanese individuals with chronic stroke who were screened initially, 28 were excluded. Finally, 30 participants were included as the analytic sample, 15 in the experimental group and 15 in the control group (Fig. 5). Participants’ demographic and clinical characteristics are listed in Table 1. The median age was approximately 60 years in both groups; the median body mass index (BMI) was higher in the control group (23.5 vs. 22.0 kg/m^2^), and the median stroke duration was longer in the experimental group (2.5 vs. 1.5 years); however, no significant between-group differences were observed (all p > 0.05).

**Table 1:** Baseline characteristics of study population

	Control	Experimental	p-value
	N = 15	N = 15	
	Median (IQR)		
Age, years	60 (52–68)	61 (53–68)	0.68^a^
BMI, kg/m^2^	23.53 (22.22–28.52)	22.03 (21.48–24.49)	0.20^a^
Duration of stroke, years	1.5 (1.08–2.33)	2.5 (1.08–5.17)	0.23^a^

	Control	Experimental	p-value
	N (%)		
Gender			
Female	6 (40%)	6 (40%)	1.00^b^
Male	9 (60%)	9 (60%)	
Side of stroke			
Left	7 (46.67%)	9 (60%)	0.46^b^
Right	8 (53.33%)	6 (40%)	
Type of stroke			
Hemorrhagic	6 (40%)	7 (46.67%)	0.71^b^
Ischemic	9 (60%)	8 (53.33%)	
Br. stage (U/E)			
II	0 (0%)	2 (13.33%)	0.06^c^
III	3 (20%)	6 (40%)	
IV	10 (66.67%)	3 (20%)	
V	2 (13.33%)	4 (26.67%)	
Br. stage (L/E)			
III	4 (26.67%)	3 (20%)	0.80^c^
IV	8 (53.33%)	7 (46.67%)	
V	3 (20%)	5 (33.33%)	

*IQR* interquartile range, *BMI* body mass index, *Br. stage* Brunnstrom stage, *U/E* upper extremity, *L/E* lower extremity

^a^Mann–Whitney U test; ^b^Chi-square test; ^c^Fisher’s exact test

The participants of both control and experimental groups were highly compliant with the assigned rehabilitation, and none dropped out of the study. Moreover, no falls or adverse effects were observed during the entire study period.

Table 2 presents the distribution of outcome measures. Differences between the two groups did not reach significance in all outcome measures in the pre-intervention and post-intervention (all p > 0.05). However, participants’ BBS scores improved significantly in both groups (control group: p = 0.01, effect size = 0.49; experimental group: p = 0.01, effect size = 0.70) (Table 2). Experimental group participants’ TUG scores improved significantly (p = 0.005, effect size = 0.70) (Table 2).

**Table 2:** Distribution of outcome measurements before and after intervention

	Control	Experimental	p-value^a^
	N = 15	N = 15	
	Median (IQR)		
BBS			
Pre-intervention	42 (32–49)	45 (32–49)	0.90
Post-intervention	46 (32–41)	50 (33–52)	0.59
p-value^b^	0.01*	0.001*	
TUG			
Pre-intervention	24.83 (16.97–57.59)	26.22 (10.7–48.56)	0.51
Post-intervention	20.22 (15.13–53.19)	23.87 (9.31–46.09)	0.51
p-value^b^	0.28	0.005*	
MFES			
Pre-intervention	126 (76–140)	90 (60–110)	0.09
Post-intervention	130 (73–140)	100 (50–122)	0.15
p-value^b^	0.50	0.77	
MI (LE)			
Pre-intervention	76 (76–84)	70 (34–76)	0.11
Post-intervention	78 (76–92)	76 (39–84)	0.20
p-value^b^	0.24	0.06	
FAC			
Pre-intervention	4 (3–4)	4 (3–4)	0.80
Post-intervention	4 (3–4)	4 (3–4)	0.59
p-value^b^	1.00	0.50	

*IQR* interquartile range, *BBS* Berg Balance Scale, *TUG* Timed Up and Go test, *MFES* Modified Falls Efficacy Scale, *L/E* lower extremity, *FAC* functional ambulation categories

^a^Mann–Whitney U test; ^b^ Wilcoxon signed-ranks test

^*^Indicates significant difference, p < 0.05

Repeated measures ANOVA was applied to evaluate the effects by group and time; results indicated that the group exerted no significant effects for all outcome measures (all p > 0.05, Additional file 1: Table S1).

## Discussion

Results of the present pilot study have demonstrated that the efficacy of the therapist-monitored, Kinect-based, interactive telerehabilitation system is similar to that of conventional therapist-delivered face-to-face therapy to improve balance in those with chronic stroke. Participation in 40-min interactive telerehabilitation exercise sessions for four weeks improved the scores of BBS and TUG. Higher BBS scores indicate greater independence and better balance ability, and shorter times to complete the TUG test correlate with lower risk of falling. Moreover, patients’ compliance and safety observed in this pilot study suggested the feasibility of interactive telerehabilitation system for stroke survivors..

The easy-to-use nature of the present exergaming system is similar to that of a home-based system reported previously by Dodakian et al. [27]. Those authors emphasized that their home-based system for upper limb training did not require users to have computer skills; the system used in the present study also did not require participants to have computer skills; they were able to easily follow the avatars on the monitor in the different exercises. The rigor of the telerehabilitation system does not appear to be compromised by the instability of individuals with chronic stroke. Many virtual reality/exercise games have been specifically designed for stroke rehabilitation, so the potential instability of stroke patients has already been considered when developing the games [23, 44, 45]. The level of difficulty of the exercises can be adjusted by the controlling therapist empirically based on patients’ individual functional status and ability to perform them. At the same time, considering motor skill learning, larger doses (longer therapeutic time or high frequency of repetition) of practice are expected to result in greater effectiveness of practice. A previous study with similar exercise intensity to the present study reported that four-weeks of home-based upper extremity gaming therapy improved upper limb function in patients with chronic stroke [46].

Hiengkaew et al. [47] suggested that the minimal detectable change (MDC) of the BBS score for individuals with chronic stroke is around 4 to 6 BBS points. In the present study, BBS scores improved significantly in the conventional physiotherapy and telerehabilitation groups, increasing by 4 and 5 points, respectively (Table 2); however, whether the magnitudes of increase in BBS scores are clinically meaningful warrants further investigation. Moreover, TUG test times were also improved significantly in the experimental group after participants completed the telerehabilitation program, although the change between before and after intervention was 2.35s less than the MDC for TUG (approximate 3–8s) [47, 48]. In contrast, TUG times were not significantly different between pre- and post-intervention in the control group, but the difference (4.61s) was with the range of the corresponding MDC. According to Cohen’s classification of effect size [49], the above-mentioned three significant changes had medium effect size. Hence, the clinical significance of the current TUG results remained to be explored. A recent study by Bower et al. [50] also demonstrated that dynamic balance measures (e.g., TUG) were more strongly predictive of falls than static balance variables in stroke survivors. However, further investigation is needed to determine whether the present target-oriented telerehabilitation system can reduce falls in individuals with stroke.

Three types of telerehabilitation programs were used in the present study: a target-oriented stepping task, multidirectional reaching task, and Tai Chi exercises—which together improved balance ability and functional mobility in individuals with chronic stroke. Weight transfer or loading/unloading ability of the paretic limb and healthy lower extremity is essential for patients with stroke and can be trained by stepping exercises [51]. Both visual and auditory feedback were used in the target-oriented stepping task to enhance participants’ balance, strength, and mobility functions. Similarly, the combination of a VR-based stepping exercise with conventional rehabilitation enhanced the balance and gait speed of individuals with chronic stroke [52]. In another pilot study, Cho et al. [53] also indicated that a virtual walking training program enhanced BBS and TUG performance in those with chronic stroke, suggesting that the locomotion of those with stroke in multiple directions benefits from focused training. A recent review demonstrated that stepping in different directions as a basis for rehabilitation strategies improves the mobility and balance ability of stroke patients [54]. The slow and rhythmic movements of Tai Chi promote physiological functions, including balance, shifting body weight from one leg to the another, relaxation, mitigating inner stress, coordination, as well as maintaining the head, shoulders, trunk, hips, and legs in alignment during movement [55]. Gatts et al. [56] reported that Tai Chi improved ankle joint control in perturbed gait in older adults with impaired balance. Another recent study also revealed that Tai Chi improved balance ability and functional mobility in individuals with stroke [57]. The entire interactive exergaming system depends upon the Kinect camera-based capabilities as described previously [58, 59].

Virtual rehabilitation can improve motor, cognitive and functional outcomes in adults who survive stroke, particularly when provided as a target-oriented program [23]. Although the physiological and neural mechanisms underlying the functional benefits of VR and exergames remain to be investigated, repeated accurate performance can be incorporated into VR and exergaming to enhance motor learning; as such, VR represents a distinctive experimental platform for understanding complex skills learning, and it should enable transfer from virtual environments to the real world [45, 60]. Training that involves playing commercial video games results in obvious gray matter increases in the right hippocampal formation, right dorsolateral prefrontal cortex, and bilateral cerebellum area [61, 62]. Calabrò et al. [63] also demonstrated that robot-assisted gait training combined with VR therapy activated the premotor, precuneus, and associative visual areas to a greater extent compared with robot-assisted gait training without VR. Regarding the effect of VR on balance and gait in persons with stroke, previous studies have applied similar approaches as the present study [64, 65]. Two previous studies found that the effectiveness was comparable between virtual reality (VR) and conventional rehabilitation [66, 67] similar to our findings with regard to telerehabilitation vs. conventional therapy.

## Strengths and limitations

The strengths of the present study include that the telerehabilitation system enabled bidirectional communication between therapists and patients with stroke. Both therapists and patients simultaneously faced screens and interacted with programs. On the therapists’ side, therapists were able to monitor participants’ exercise situation in the remote area. Therapists could also monitor multiple applications simultaneously, enabling therapists to observe patients’ status more efficiently and reduce related costs. On the end-user side, the scores and duration of rehabilitation were displayed on the screen. Hence, individuals with chronic stroke had real-time and automatic visual feedback on how well they were executing the exercises.

The present pilot study also has several limitations, including the small sample size, as well as short intervention duration, which precludes long-term evaluation. Also, the telerehabilitation was performed in an independent room in the hospital rehabilitation department and was not actually performed in a home setting as intended for future use. Instead, participants underwent autonomous rehabilitation in an independent space in the hospital rehabilitation department while family or caregivers stood beside them to provide assistance and to prevent falls. Another limitation is that we did not investigate differences in results between stroke sides, which will be included in future studies. In addition, we have to admit that there is still room for improvement of telerehabilitation program used in the telerehabilitation system; more optimal exercise programs based on motor learning principles (e.g. feedback, task-specificity and optimal level of challenge) have to be developed specifically for stroke survivors. Moreover, further prospective study is needed to evaluate the system in a home-based remote setting, while also expanding the study sample and the intervention period.

## Conclusions

An interactive telerehabilitation system using the newly designed Kinect-based exergaming system allows therapists to remotely monitor the rehabilitation exercises of individuals with chronic stroke. Using target-oriented exercise programs, the telerehabilitation system demonstrated either superior or equal efficacy compared with conventional one-on-one physiotherapy for individuals with chronic stroke. Results of the present pilot study may pave the way for continued development of home-based interactive telerehabilitation in the future.

## Supplementary Information

**Additional file 1: Table S1.** Repeated measures ANOVA of outcome measurements.

## Data Availability

The datasets generated during the present study are available from the corresponding author upon request.

## Abbreviations

BBB: Berg balance scale
FAC: Functional ambulation category
MFES: Modified falls efficacy scale
MI: Motricity index
TUG: Timed up and go
SD: Standard deviation
