# Supplementary material for: Feasibility and effect of interactive telerehabilitation on balance in individuals with chronic stroke: a pilot study
Source: J Neuroeng Rehabil. 2021 Apr 26;18:71. doi: 10.1186/s12984-021-00866-8 (PMC8077813; doi:10.1186/s12984-021-00866-8)
Supplement: Supplementary file 1 — Additional file 1: Table S1. Repeated measures ANOVA of outcome measurements. [file 12984_2021_866_MOESM1_ESM.docx]

Supplemental Table 1. Repeated measures ANOVA of outcome measurements

|  | F statistic | p-value |
| --- | --- | --- |
| BBS |  |  |
| Group | 0.06 | 0.81 |
| Time | 28.9 | <0.0001* |
| Group*Time | 0.21 | 0.65 |
| TUG |  |  |
| Group | 0.92 | 0.35 |
| Time | 1.94 | 0.18 |
| Group*Time | 0.83 | 0.37 |
| MFES |  |  |
| Group | 2.37 | 0.14 |
| Time | 0.19 | 0.66 |
| Group*Time | 0.01 | 0.93 |
| MI |  |  |
| Group | 2.05 | 0.16 |
| Time | 5.33 | 0.03* |
| Group*Time | 0.08 | 0.78 |
| FAC |  |  |
| Group | 0.07 | 0.79 |
| Time | 3.15 | 0.09 |
| Group*Time | 0.35 | 0.56 |

BBS: Berg Balance Scale; TUG: Timed Up and Go test; MFES: Modiﬁed Falls Efﬁcacy Scale; FAC: functional ambulation categories Results are shown as P values.

^*^indicates significant difference, p<0.05.
